# Heterogeneity of Cardiovascular Response to Standardized Sepsis Resuscitation

**DOI:** 10.1186/s13054-020-2779-9

**Published:** 2020-03-24

**Authors:** Fabio Guarracino, Pietro Bertini, Michael R. Pinsky

**Affiliations:** 1 Department of Anesthesia and Critical Care Medicine, Azienda Ospedaliero Universitaria Pisana, Pisa, Italy; 2 Department of Critical Care Medicine, University of Pittsburgh Medical Center, Pittsburgh, PA USA

## Abstract

This article is one of ten reviews selected from the Annual Update in Intensive Care and Emergency Medicine 2020. Other selected articles can be found online at https://www.biomedcentral.com/collections/annualupdate2020. Further information about the Annual Update in Intensive Care and Emergency Medicine is available from http://www.springer.com/series/8901.

## Introduction

The Surviving Sepsis Campaign (SSC) guidelines [1] recommend a hemodynamic optimization strategy to rapidly counteract the impact of sepsis on blood flow in the first few hours after diagnosis. Specifically, the SSC guidelines suggest promptly restoring and ameliorating circulatory shock using early and aggressive volume expansion with crystalloids (30 ml/kg) to achieve a mean arterial pressure (MAP) of at least 65 mmHg. If this initial volume expansion fails to restore MAP, then clinicians are allowed to use vasopressor agents and subsequently inotropic support to achieve this goal. This standardized and mono-dimensional approach to cardiovascular stabilization flies in the face of numerous clinical observations. For example, a large database analysis of patients with septic shock (*n* = 3686) consistently reported that only two-thirds of patients were volume responders [2]. Patients not responding to volume expansion may experience fluid overload, which is in and of itself an independent risk factor for prolonged hospitalization, death, and poor outcome as previously described [3].

### Physiologic Rationale for Resuscitation

The physiologic rationale for initial volume resuscitation in the septic hypotensive patient is not straightforward, although fluid resuscitation is often effective in restoring MAP. The only thing that volume expansion can do is increase circulating blood volume and, by inference, mean systemic pressure (*P*_ms_), which is the upstream pressure driving venous return. *P*_ms_ is a function of the relation between stressed blood volume and vascular compliance. Total blood volume is distributed across the vascular space into volume that does not increase *P*_ms_, referred to as unstressed volume, and volume that does cause *P*_ms_ to increase. Under normal resting conditions, approximately 60–70% of the total circulating blood volume is unstressed volume with a majority of that volume in the splanchnic circulation. Increasing sympathetic tone and exercise decrease splanchnic blood flow distributing more of the blood volume to vascular spaces with lower unstressed volume, thereby increasing *P*_ms_. Furthermore, for the associated increase in *P*_ms_ to increase cardiac output, the right ventricle needs to be volume responsive as manifest by an associated increase in the right atrial pressure to *P*_ms_ gradient, because venous return can only increase if this gradient increases, the resistance to venous return decreases, or both occur. Finally, in a fluid-responsive septic patient, for MAP to also increase in parallel to the increase in cardiac output, the arterial vasomotor tone must be sufficient to realize an associated increase in pressure to follow the increase in flow. What is unclear is how these processes play out in individual patients presenting with hypotensive sepsis.

**Fig. 1:**
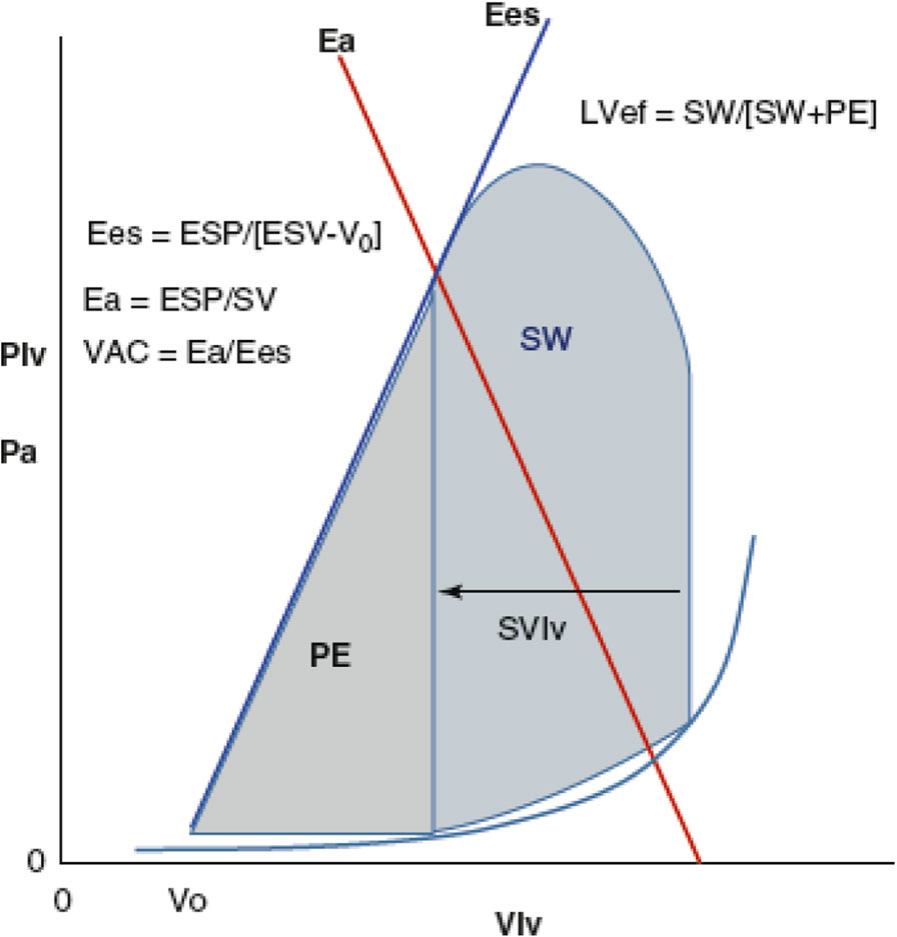
A stylized representation of the relation between left ventricular (LV) pressure (*P*_lv_) or arterial pressure (*P*_a_) and LV pressure-volume relations during a cardiac cycle and arterial elastance (*E*_a_) (red line) along with the associated formulae defining end-systolic elastance (*E*_es_) (blue line) and *E*_a_. Stroke work (SW) is the area within the LV pressure-volume loop for one cardiac cycle, while the potential energy (PE) is the area sub-served by the *E*_a_ and LV end-systolic volume (ESV). LV efficiency (LVef) is the ratio of SW to SW + PE (Reproduced from [5] under a Creative Commons Attribution 4.0 International License)

Coupled with the effects of sepsis on the systemic circulation is the interaction between left ventricular (LV) pump function and its arterial load, referred to as ventriculoarterial coupling. Ventriculoarterial coupling is characterized by the relation between LV elastance (*E*_es_), the primary parameter defining LV contractility, and effective arterial elastance (*E*_a_), a clinical surrogate of LV afterload [4]. The determinants of these LV performance parameters are summarized in Fig. 1.

### Clinical Observations

We recently demonstrated in a cohort of 55 septic hypotensive patients that a majority reversed their hypotension in response to volume expansion alone [5]. Since septic shock is defined as hypotension not responsive to volume expansion alone, most patients in our cohort had sepsis but not septic shock. Analyzing the physiologic determinants further, we found that most septic shock patients with hypotension despite volume expansion, owing to loss of arterial tone (i.e., vasoplegia), also displayed significant alterations in ventriculoarterial coupling. The majority of our septic shock patients displayed significant ventriculoarterial uncoupling, with *E*_a_ markedly greater than *E*_es_. Ventriculoarterial uncoupling markedly decreases LV ejection efficiency and can independently lead to heart failure [4].

In our recent observational study [5] on the effect of therapies on the determinants of cardiovascular status as recommended by the SSC guidelines, we confirmed the efficacy of volume expansion but the results cast light on the lack of knowledge about timing and appropriate sequence of hemodynamic resuscitation following volume expansion and vasoactive and inotropic agents. In patients with elevated baseline *E*_a_ for example, poor hemodynamic performance was seen after treatment with norepinephrine with less improvement in MAP or cardiac output [5].

In summary, these findings collectively underscore the heterogeneity of cardiovascular responses to a SSC guideline-defined resuscitation protocol in septic patients owing to similarly heterogeneous pathophysiologic states.

In most of our patients, volume expansion was able to restore MAP to >65 mmHg by also increasing cardiac output, *E*_es_ and *P*_ms_, leading to improved energy transfer measurements, such as LV ejection efficiency, ventriculoarterial coupling, and heart efficiency. Interestingly, we documented that volume expansion also increased *E*_es_. *E*_es_ is considered a load-independent measurement of LV contractility. Our observed increase in *E*_es_ was probably due to the restoration of coronary perfusion pressure demonstrated by MAP increase.

Importantly, we found that individual patient MAP and cardiac output responses to volume expansion were variable, but accurately predicted by baseline pulse pressure variation (PPV), stroke volume variation (SVV) and their ratio, and dynamic elastance (Ea_dyn_), suggesting that fluid resuscitation based on these dynamic measures may be more efficient by resulting in less fluid being given to nonresponders [5]. This is also consistent with prior findings that PPV predicts cardiac output responses to volume expansion in septic patients [6] and that Ea_dyn_ predicted the associated change in MAP in response to changing cardiac output [7]. These data also support the clinical relevance of using functional dynamic measurements to tailor fluid administration in septic patients as recommended by the SSC guidelines [1].

Several studies describe a variable response to norepinephrine in septic shock [8]. In our investigation [5], norepinephrine increased *E*_a_ and MAP in most patients but did not achieve a MAP >65 mmHg in the majority and induced ventriculoarterial uncoupling to levels seen prior to resuscitation; it also decreased LV ejection efficiency, which, if sustained, might impair LV performance. These data support the recent observation that sustained use of vasopressors for >6 h in septic shock to maintain a MAP >75 mmHg is associated with increased mortality [9]. Recently, it has been shown in animal studies that norepinephrine can impair LV ejection by increasing the magnitude of arterial pressure reflected waves during ejection, which also becomes manifest as ventriculoarterial uncoupling without increasing coronary perfusion pressure [10]. As was also seen in patients with postoperative vasoplegia [11], we observed that only patients with higher *E*_es_ and normalized ventriculoarterial coupling increased cardiac output during norepinephrine infusion, presumably because they can tolerate the increased afterload [5].

When dobutamine was added to volume expansion and norepinephrine in a few patients, it restored normal ventriculoarterial coupling and cardiac output, suggesting that inotropic support may improve contractility in septic patients who may be affected by septic cardiomyopathy [12]. These findings have also been reported by others [4, 11, 13]. Analyzing these norepinephrine- and dobutamine-dependent subsets in future investigations may improve our understanding of how vasoactive and inotropic therapies change hemodynamics [8].

### Clinical Relevance

Potentially, the selection of the most appropriate treatment in septic shock patients following initial volume expansion could be ascertained by knowing their *E*_a_, *E*_es_ and dynamic parameters, such as Ea_dyn_. Similarly, volume expansion should be individualized based on dynamic measures of volume responsiveness.

**Fig. 2:**
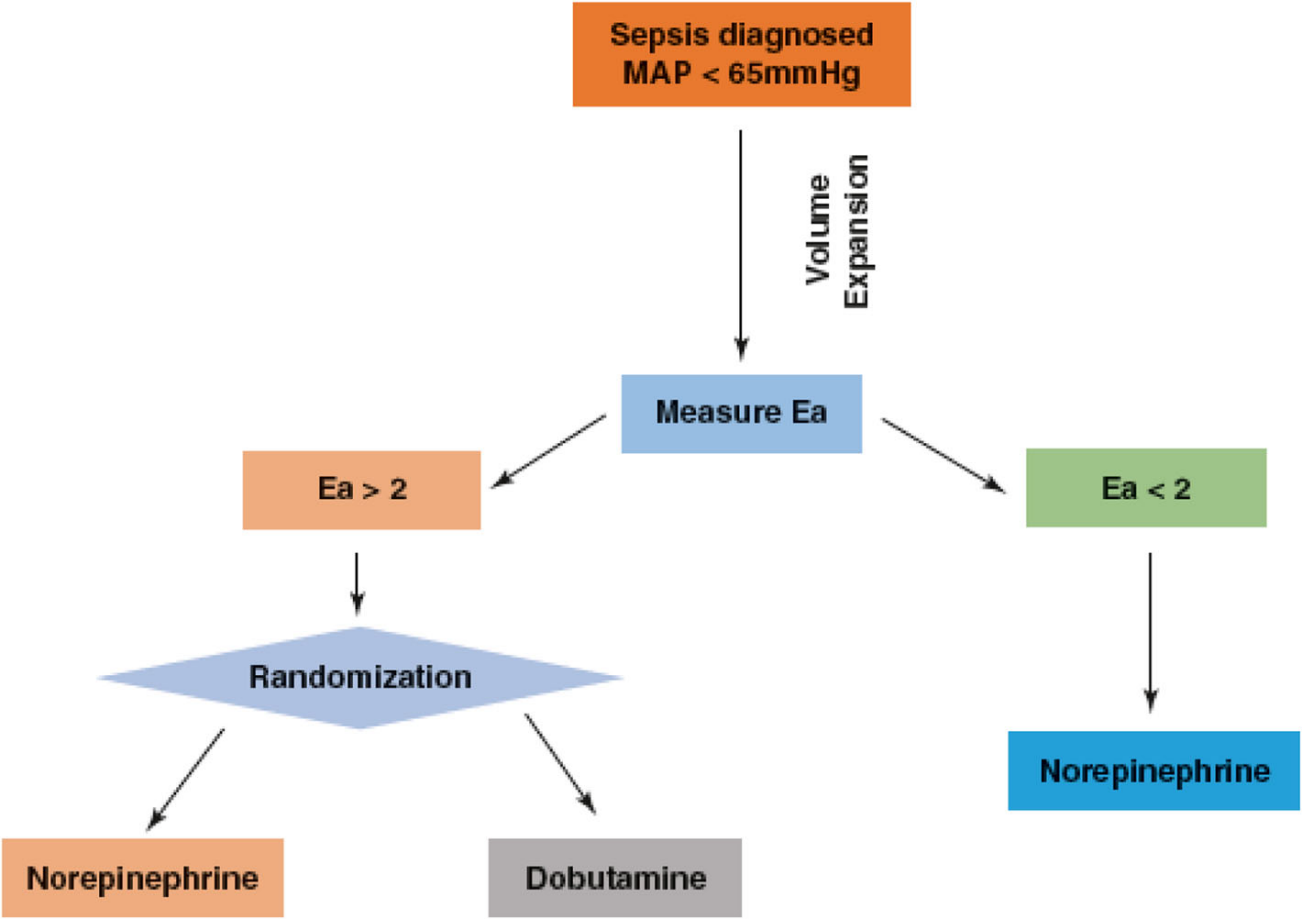
A proposed treatment algorithm to personalize resuscitation in septic patients. *E*_*a*_ arterial elastance

We suggest that a prospective clinical trial could be conducted to address this specific approach. The criterion for patient recruitment would be the same as for previous investigations, i.e., sepsis with MAP <65 mmHg. Since an initial volume expansion step was beneficial in the majority of patients, it would be the initial treatment but given based on the functional dynamic parameters, PPV and SVV. In patients who do not achieve MAP >65 mmHg after volume expansion, *E*_a_ would be measured. If *E*_a_ were <2 mmHg/ml, patients would receive norepinephrine aimed at achieving a MAP >65 mmHg. If *E*_a_ were ≥2 mmHg/ml, patients would be randomly allocated to either norepinephrine or dobutamine (Fig. 2) in order to verify the beneficial effect of an early inotropic strategy versus a vasoconstrictive one.

## Conclusion

The determinants of the cardiovascular state collectively called sepsis and septic shock are complex and heterogeneous. The response to resuscitation is also heterogeneous, and thus treatment needs to be individualized to maximize timeliness of appropriate therapies while avoiding volume overload. Prospective clinical trials will help illuminate the optimal strategies, but the principle of individualizing treatment is already valid.

## Data Availability

Not applicable.
